# Systemic Delivery of hGhrelin Derivative by Lyophilizate for Dry Powder Inhalation System in Monkeys

**DOI:** 10.3390/pharmaceutics13020233

**Published:** 2021-02-07

**Authors:** Kahori Miyamoto, Yuko Ishibashi, Tomomi Akita, Chikamasa Yamashita

**Affiliations:** Department of Pharmaceutics and Drug Delivery, Faculty of Pharmaceutical Sciences, Tokyo University of Science, 2641 Yamazaki, Noda, Chiba 278-8510, Japan; 3A17706@ed.tus.ac.jp (K.M.); j3a08010@ed.tus.ac.jp (Y.I.); akitat@rs.tus.ac.jp (T.A.)

**Keywords:** hGhrelin derivative, dry powder inhalation, systemic delivery, monkey, pulmonary administration

## Abstract

Ghrelin is the peptide that increases the hunger sensation and food intake and is expected to be clinically applied for treatment of diseases such as cachexia and anorexia nervosa. In the clinical application of ghrelin, injections are problematic in that they are invasive and inconvenient. Thus, we aimed to develop a formulation that can eliminate the need for injections and can be applied clinically. We prepared formulations of an hGhrelin derivative, in which the octanoyl group essential for expression of activity is modified to avoid rapid des-acylation, using lyophilizate for a dry powder inhalation (LDPI) system. The formulation of hGhrelin derivative was optimized by the addition of phenylalanine, of which the fine particle fraction of 5 µm or less was 41.7 ± 3.8%. We also performed pharmacokinetic/pharmacodynamic tests in monkeys using the optimum formulation that can be applied clinically. The absolute bioavailability of inhaled hGhrelin derivative with respect to that intravenously injected was 16.9 ± 2.6%. An increase in growth hormone was shown as an effect of the inhaled hGhrelin derivative similar to intravenous injection. The LDPI formulation can deliver the hGhrelin derivative systemically, and it is expected to be applied clinically as a substitute for injections.

## 1. Introduction

Ghrelin is the only currently known peripherally produced peptide that increases hunger sensation and food intake [1,2,3]. Ghrelin was first isolated from rat and human stomachs in 1999 as a growth hormone secretagogue (GHS) [4]. Ghrelin is an endogenous ligand and acts through the GHS-receptor (GHSR) [4,5]. Ghrelin having a similar primary structure was isolated not only from rat and human but also from many vertebrates such as mouse and bullfrog [6,7,8,9]. Human ghrelin (hGhrelin) is a peptide hormone of 28 amino acids, and its activity is expressed by *O*-*n*-octanoylation at the 3rd serine residue [4,6,10]. Ghrelin is a multifaceted peptide hormone [6,11]. Effects on growth hormone (GH) release and appetite are well known as effects of ghrelin that are involved in the regulation of food intake and energy balance [5,6,12,13]. Ghrelin increases food intake by stimulation of the appetite through the orexigenic neuropeptide Y/agouti-related protein neurons of the arcuate nucleus in the hypothalamus [14,15]. Because of its anabolic effects (stimulation of appetite, adiposity, and blood glucose), ghrelin is expected to be clinically applied for treatment of diseases such as cachexia and anorexia nervosa [16,17,18], and clinical trials for the treatment of eating disorders and cachexia with rapid intravenous injection of ghrelin were conducted [19,20]. Long-term administration of ghrelin is necessary for treatment of its target diseases such as cachexia and eating disorders. Therefore, development of less stressful formulations of ghrelin is desirable. Peptide drugs are predominantly administered by either intravenous or subcutaneous injection, but parenteral injection is not the preferred route of administration due to pain, risk of infection, and low patient compliance in general [21,22,23]. Moreover, safety issues related to the disposal of needles discourage parenteral administration [22]. Most people with eating disorders and cachexia receive their treatment in an outpatient setting [24]. Injections are likely to require frequent hospital visits leading to increased burden on patients. Especially in regard to eating disorders, resistance to treatment and the high rate of dropouts from treatment are problems [25,26,27,28,29,30]. The disadvantages of injections decrease motivation for treatment, and thus, injections are not preferable for treatment of eating disorders and cachexia.

**Figure 1:**
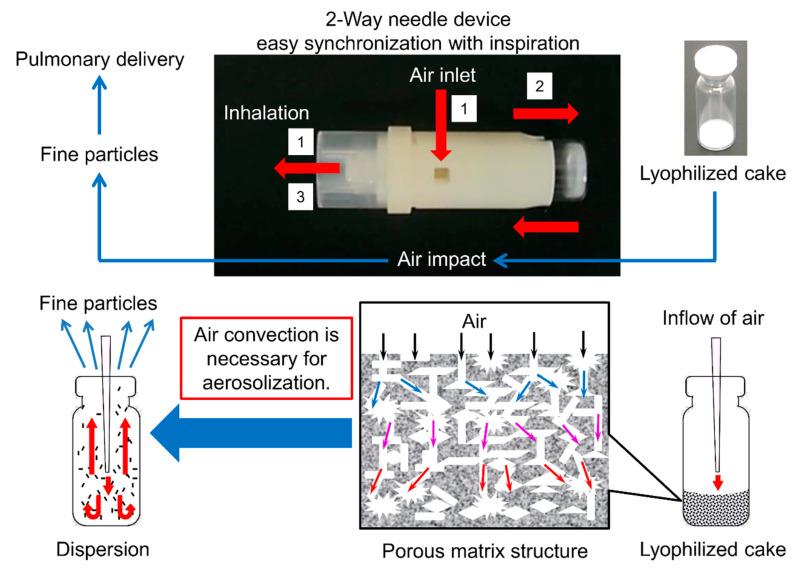
Lyophilizate for dry powder inhalation (LDPI) system. In this novel dry powder inhaler using a two-way needle device, air is introduced into the vial in synchronization with the patient’s inhalation through the two-way needle device. Then, lyophilizate with a porous matrix structure is broken into pieces by air impact and aerosolized by the convection flow of air in the vial. Finally, porous particles reconstructed from pieces of lyophilizate are emitted through the device. LDPI formulations with the two-way needle device were used for characteristic inhalation tests by multi-stage liquid impinger. LDPI formulations with another device, which is described later, were used for pharmacokinetic/pharmacodynamic tests in monkeys.

Inhalation is one of the dosage forms that can solve the problems of injections [22,31]. In the present study, we decided to develop an inhaled product as a substitute for injections based on the following: (1) The lungs have a large surface area. The total surface area of the alveoli is 100–140 m^2^, half the area of a tennis court (260 m^2^). (2) The concentration of metabolic enzymes is lower in lungs than in the gastrointestinal tract. (3) Once drugs deposit in the alveolar region, rapid drug absorption avoiding the hepatic first-pass effect occurs because the alveoli epithelium is close to the systemic circulation compared to other epithelia [32,33]. Pulmonary delivery is the only route of administration that meets the above three features; hence, inhaled products are considered to be the most preferable formulation for systemic absorption of peptides of large molecular weight that are easily biodegraded. Therefore, we focused on dry powder inhalers (DPIs) that are non-invasive and convenient from the viewpoint of both less burden on patients and better systemic absorption. Regarding the regulatory issues, tests for uniformity of delivered dose and aerodynamic particle size measurement are mainly required for inhaled products in pharmacopeias. It is difficult to say that inhaled products are strictly regulated compared to other non-injection formulations such as oral preparations or nasal products although more strict evaluations of the in vitro equivalence are required for generic inhaled products. From another viewpoint of pharmacopeias and good manufacturing practice, inhaled products are considered as more preferable dosage forms than injection. Inhaled products require less strict management than injectables because inhaled products do not fall into the realm of sterile products. However, unlike other dosage forms, inhalants are dosage forms that are established as an inhalation system that combines a formulation and an inhalation device. Currently available inhalation systems can be divided into three principal categories: nebulizers, pressurized metered-dose inhalers, and DPIs. Among these, DPIs have attracted attention due to their convenience and because of environmental considerations [33,34,35]. Dry powder formulations for inhalation are widely manufactured by jet milling or spray-drying. However, peptides and proteins are exposed to the risk of degradation and denaturation due to shear stress or high temperature during the manufacturing process using these methods [36,37,38]. Thus, we focused on the system developed by Yamashita et al. [39,40,41,42,43,44], termed lyophilizate for dry powder inhalation (LDPI). In LDPI system, a freeze-dried cake with a porous matrix structure is broken into particles suitable for pulmonary administration by the impact of air introduced in synchronization with the patient’s inspiration (Figure 1). LDPI formulations are prepared by a very simple method of lyophilization in which a glass vial is filled with the preparation solution that is then freeze-dried. Therefore, LDPI system is expected to enable pulmonary formulations of heat-sensitive drugs such as peptides and proteins [42,43,45].

Ghrelin is known to be easily des-acylated in blood plasma because the ester bond is easily cleaved [46,47], although acylation at the 3rd serine residue is necessary for expression of activity. Therefore, we focused on the hGhrelin derivative [48,49]. The ester bond or the lipidic regions of hGhrelin derivative are modified (Figure S1) so that it is less likely to be des-acylated [48,50,51].

From this background, we aimed to develop a clinical powder inhalation formulation of hGhrelin derivative for use with the LDPI system that can solve the problems of injections. We also conducted pharmacokinetic/pharmacodynamic tests in monkeys using the developed LDPI formulation, which can be used clinically to evaluate its feasibility for clinical application.

## 2. Materials and Methods

### 2.1. Materials

hGhrelin derivative was obtained from Asubio Pharma (Kobe, Japan). The following six amino acids (special grade)—l-alanine (Ala), l-methionine (Met), l-phenylalanine (Phe), l-leucine (Leu), l-valine (Val), and l-isoleucine (Ile), 1 mol/L hydrochloride (HCl, special grade), benzalkonium chloride, acetic acid (for volumetric analysis), acetonitrile (HPLC [high-performance liquid chromatography] grade), trifluoroacetic acid (TFA, HPLC grade), disodium hydrogen phosphate (special grade), sodium dihydrogen phosphate (special grade), sodium chloride (special grade), sodium azide (special grade), and albumin from bovine serum (BSA, for biochemistry) were all purchased from Fujifilm Wako Pure Chemical Industries (Osaka, Japan). Isoflurane was purchased from Mylan Seiyaku Ltd. (Tokyo, Japan). Disodium ethylenediaminetetraacetic acid dihydrate (EDTA, special grade) was purchased from Dojindo Laboratories (Kamimashiki, Japan). Triton^®^-X was purchased from Nacalai Tesque (Kyoto, Japan). Pefabloc^®^ SC (4-(2-aminoethyl)benzenesulfonyl fluoride) was purchased from Roche Applied Sciences (Penzberg, Bayern, Germany). Isotonic sodium chloride solution (saline) was purchased from Otsuka Pharmaceutical (Tokyo, Japan).

The packaging materials used in this study were obtained from the following commercial vendors: 2-mL VIST glass vials from Daiwa Special Glass (Osaka, Japan) and rubber stoppers (F5-43) from Sumitomo Rubber Industries (Kobe, Japan).

The catheter (SAFEED^®^ Nelaton catheter with adaptor 14 Fr) was purchased from Terumo (Tokyo, Japan).

### 2.2. Preparation of Freeze-Dried Cake for Inhalation of hGhrelin Derivative

Stock solutions of amino acids were prepared by dissolving them in purified water (10 mg/mL). hGhrelin derivative was dissolved in purified water, and then stock solutions of the amino acids were added. These solutions containing hGhrelin derivative and amino acids were diluted with purified water to obtain the target concentration. HCl at 1 mol/L was added to the diluted solution to adjust pH to 3–4. Then, 500 μL of the solution was added to a vial and lyophilized by a benchtop freeze dryer (FreeZone Triad 7400030, LABCONCO, Kansas City, MO, USA). The lyophilization conditions were as follows. Shelf cooling was performed from room temperature to −70 °C (≤0.5 °C/min), and shelf temperature was held at −70 °C for 3 h. Primary drying was performed at a shelf temperature of −30 °C and pressure of 1.0 Pa for 11 h. In the secondary drying step, shelf temperature was ramped to 35 °C (0.17 °C/min) for 5 h and then decreased to 25 °C (0.34 °C/min) for 1 h.

### 2.3. Quantification of hGhrelin Derivative by HPLC

HPLC (Prominence series, Shimadzu, Kyoto, Japan) was used to quantify the hGhrelin derivative according to a previous study [52]. Sample solution was applied in a TSKgel ODS-80Ts column (150 × 4.6 mm, 3 µm; TOHSO, Tokyo, Japan) at a temperature of 40 °C and flow rate of 1.0 mL/min. The concentration gradient of the mobile phase was controlled as follows: from 90% mobile phase A (0.1% aqueous solution of TFA) and 10% mobile phase B (0.085% TFA in acetonitrile); 10 min, 24% mobile phase B; 25 min, 30% mobile phase B; 26–29 min, 70% mobile phase B. hGhrelin derivative was detected by UV absorption photometer at a wavelength of 214 nm. The limits of detection and quantitation were 0.1 and 1.5 µg/mL, respectively.

### 2.4. Visual Evaluation of Cake Appearance

In lyophilized formulations, the lyophilized cake is often required to have a good appearance without cracks or chips [53]. In the LDPI system, a freeze-dried cake with good appearance is necessary to ensure stable performance. Thus, the cake appearance was evaluated by configuration score.

The freeze-dried cake was classified into three types according to appearance: configuration score 0, 1 or 2. Configuration score 0 indicates that no cake was formed. Configuration score 1 indicates that the appearance of the cake was not suitable because cracks or chips were observed. Configuration score 2 indicates that a cake without cracks or chips was formed and that its appearance was suitable for the LDPI formulation.

### 2.5. Particle Size Distribution

It is ideal to evaluate all samples by inhalation characteristic tests, but such tests require much time even for only a few samples. In screening studies, we measured the particle size distribution by aerodynamic particle sizer (APS) to optimize formulations in a shorter time. The result of APS was used as a surrogate benchmark for inhalation characteristic tests by multi-stage liquid impinger (MSLI).

#### 2.5.1. Aerodynamic Particle Size Distribution by APS Spectrometer

**Figure 2:**
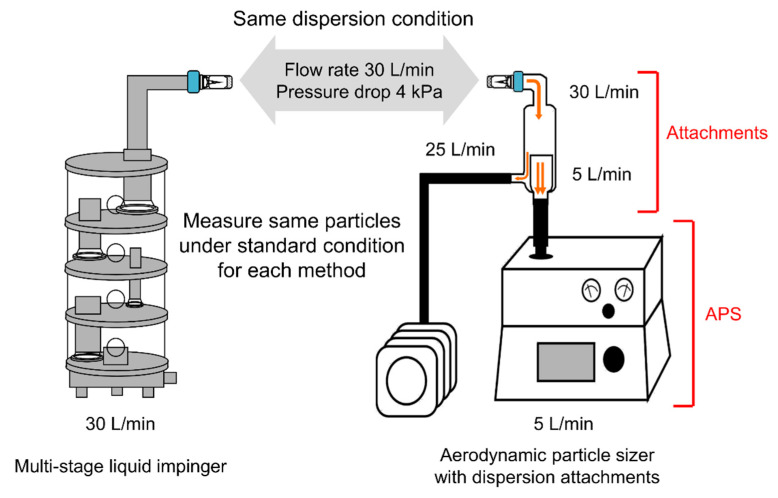
Methods to measure the aerodynamic particle distribution; multi-stage liquid impinger (MSLI) and aerodynamic particle sizer (APS) with dispersion attachments. Formulations are dispersed under the same condition as for MSLI measurement (flow rate of 30 L/min at a pressure drop of 4 kPa with the two-way needle device). Dispersed particles are measured under the standard condition for each method. In the MSLI, the aerodynamic particle size distribution is measured at a flow rate of 30 L/min by mass. In the APS, the aerodynamic particle size distribution is measured at a flow rate of 5 L/min based on time-of-flight theory.

We have developed a measurement method using APS in which the APS results can be used to estimate the MSLI results [44], and APS measurement was performed by this method (Figure 2). Briefly, to set the dispersion condition for APS measurement to match that for MSLI measurement, a custom-made glass throat was placed on top of the aerosol diluter of the APS (flow rate 5 L/min; Model 3302A, TSI, Shoreview, MN, USA), and a linear vacuum pump (flow rate adjusted to 25 L/min; VP0940, Nitto Kohki, Tokyo, Japan) was added. The freeze-dried cake was micronized under the above conditions, which are the same conditions as for MSLI measurement. The particle size distribution was measured every 1 s for 8 s with a model 3321 APS spectrometer (TSI). In cumulative particle size distribution, the percentage of fine particles of 5 µm or less was defined as APS fine particle fraction (FPF)%_≤5 μm_. Each sample was measured in triplicate.

#### 2.5.2. Inhalation Characteristic Test by Multi-Stage Liquid Impinger

Inhalation characteristic tests were performed by MSLI. The measurement by MSLI was conducted at a flow rate of 30 ± 0.3 L/min with a two-way needle device (Otsuka, Tokyo, Japan) given a pressure drop of 4 kPa. The two-way needle device used in MSLI measurement was a device in clinical use (Figure 1). A vacuum pump (HCP5), critical flow controller (TPK2000), and flow meter (DFM2000) (all from Copley Scientific Limited, Nottingham, UK) were used to maintain the above condition. A hydrophilic poly(vinylidene fluoride) membrane with a diameter of 90 mm and retention diameter of 0.65 μm (Merck Millipore, Burlington, MA, USA) was placed at stage 5 of the MSLI. Under the above condition, the freeze-dried cake was dispersed into aerosols for 8 s. Each deposition, the experiment was repeated in triplicate.

The diluent (0.01% benzalkonium chloride in 10 mM acetic acid) at each stage of the MSLI was removed for analysis. The vial, device, induction port, and membrane were each washed with the diluent. The concentration of hGhrelin derivative in the fraction recovered from the vial, the device, the induction port, and each of stages 1–5 of the MSLI was determined by HPLC.

The MSLI FPF%_≤5 μm_ was defined as the ratio of hGhrelin derivative of 5 μm or less to the emission. The proportion of hGhrelin derivative of 5 μm or less was calculated by interpolation from a plot of cumulative proportion vs. effective cut-off diameter of the respective stages (at a flow rate of 30 L/min, effective cut-off diameters for stages 2, 3, and 4 were 9.6, 4.4, and 2.4 μm, respectively). The emission was defined as the mass ratio of the emitted dose to hGhrelin derivative content of the formulation. The emitted dose was the sum of the hGhrelin derivative mass of the induction port and each of stages 1–5 of the MSLI.

For cumulative particle distribution, the aerodynamic particle size of the cumulative percentage at 50% was defined as the mass median aerodynamic diameter (MMAD).

### 2.6. Stability Tests

To study stability in the formulation process, the hGhrelin derivative content of the prepared solution before lyophilization and that of the lyophilized cake were measured by HPLC.

To study storage stability, quantification of the hGhrelin derivative by HPLC and inhalation characteristic test by MSLI were performed using formulations stored at 25 °C, 60% RH for 3 months.

### 2.7. Pharmacokinetic (PK)/Pharmacodynamic (PD) Tests

#### 2.7.1. Animals

Male cynomolgus monkeys (age 5 years 9 months–6 years 3 months; weight 5.13–5.81 kg; B virus free; from Cambodia) were studied. Animals were maintained under controlled temperature, humidity, and light conditions (25 ± 2 °C, 50 ± 10% RH, light on 07:00–19:00). All animal procedures were performed according to the protocol reviewed and approved by the Animal Care and Use Committee of TRANS GENIC INC. (No. 2012-4).

#### 2.7.2. Pulmonary Administration

**Figure 3:**
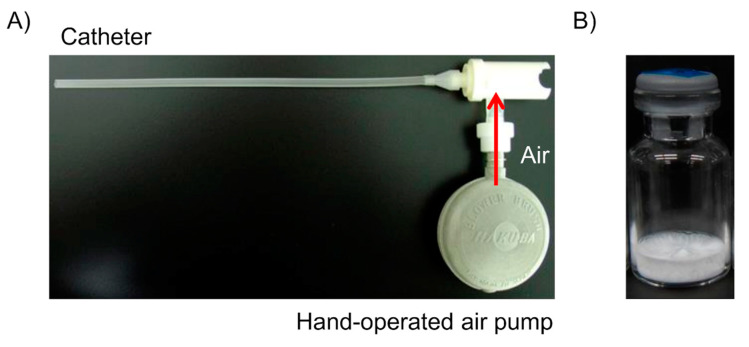
(**A**) Device used for pulmonary administration of lyophilizate for dry powder inhalation (LDPI) formulation to monkeys. (**B**) LDPI formulations containing 0.5 mg/vial of hGhrelin derivative. LDPI formulations are sprayed by hand-operated air pump and inhaled during the monkey’s inspiration through the catheter. The pulmonary administration device for monkeys was self-made and not for clinical use, whereas the formulations can be clinically used.

The monkeys were held in a monkey chair and anesthetized with inhalation anesthesia (oxygen 1.0 L/min, isoflurane 5%, 3 min) only during administration. A catheter that had been kept warm at 40 °C was inserted into the trachea, and the catheter was closed with forceps except for inhalation. A device (Figure 3A) with the formulation (Figure 3B) was connected to the catheter for administration. The formulation was sprayed by hand-operated air pump, and hGhrelin derivative was inhaled during the monkey’s inspiration. The pulmonary administration device used for monkeys (Figure 3A) was made by the authors from a two-way needle device in clinical use (Figure 1). The LDPI formulations optimized in the optimization study were administered.

For the measurement of plasma hGhrelin derivative and GH concentrations, blood samples (approximately 2 mL) were collected before and 5, 10, 20, 30, 45, 60, 90, and 120 min after the administration.

#### 2.7.3. Measurement of Plasma Concentration of hGhrelin Derivative

The concentration of hGhrelin derivative in plasma was measured by the radioimmunoassay (RIA) method according to a previous study [54]. Approximately 1.2 mL of the collected blood sample was mixed with 24 µL of 500 mmol/L Pefabloc^®^ SC solution and 12 µL of 10% EDTA saline solution and centrifuged at 4 °C at 10,000× *g* for 2 min to obtain plasma. Then, 500 µL of the obtained plasma was mixed with 50 µL of 1 mol/L HCl, and a primary antibody that recognizes the N-terminal of the hGhrelin derivative was added. The primary antibody was diluted with RIA buffer (mixture of 50 mmol/L sodium phosphate buffer [pH 7.4], 0.5% BSA, 0.5% Triton^®^-X 100, 80 mmol/L sodium chloride, 25 mmol/L EDTA, and 0.05% sodium azide) containing 0.5% standard rabbit serum. The mixture was incubated for 12 h. Subsequently, 100 μL of [^125^I] ligand solution (15,000 cpm) was added, and the mixture was incubated for 36 h. Next, 100 μL of a secondary antibody (anti-rabbit immunoglobulin G goat serum) was added, and the mixture was incubated for 24 h followed by centrifugation at 3000 rpm for 30 min. After the supernatant was removed, the radioactivity of the pellet was measured by an ARC-600 γ counter (Hitachi Aloka Medical Co., Ltd., Tokyo, Japan). The limits of detection and quantitation were 2.5 ng/mL and 14 ng/mL as the plasma concentrations, respectively.

#### 2.7.4. Measurement of Plasma Concentration of GH

The plasma concentration of GH was measured by the RIA method. Plasma was obtained from approximately 0.8 mL of the collected blood sample in the same way as described in Section 2.7.3. The concentration of GH in the obtained plasma was measured by an ARC-600 γ counter (Hitachi Aloka Medical Co., Ltd., Tokyo, Japan) using a human GH kit for blood test “Daiichi” (TFB, Tokyo, Japan).

## 3. Results

### 3.1. Optimization of Formulations

#### 3.1.1. Selection of Excipients

**Figure 4:**
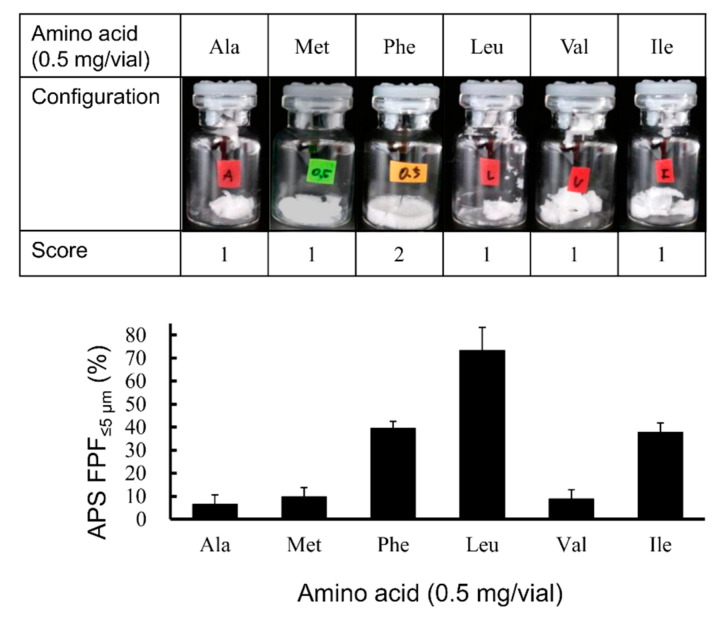
Effect of hydrophobic amino acids on configuration and fine particle fraction in the LDPI system. Representative photographs of formulations, configuration score, and values of APS FPF%_≤5 μm_ (mean ± SD, *n* = 3) are shown. Formulations were prepared to contain 0.5 mg/vial of hGhrelin derivative with 0.5 mg/vial of amino acid. APS, aerodynamic particle sizer; FPF, fine particle fraction; Ala, alanine; Met, methionine; Phe, phenylalanine; Leu, leucine; Val, valine; Ile, isoleucine.

Excipients play an important role in the generation of particles suitable for inhalation in the LDPI system [45]. In comparison to saccharides, which are widely used as excipients for freeze-dried preparations, amino acids (especially hydrophobic amino acids) showed high aerosolization performance as excipients for LDPI formulations [39,45,55]. Therefore, we performed the screening study focusing not on saccharides but on hydrophobic amino acids. Visual evaluation of the cake appearance and APS measurement were performed using formulations containing 0.5 mg/vial of hGhrelin derivative and hydrophobic amino acid, for which the hydropathy index is a positive value (isoleucine: 4.5, valine: 4.2, leucine: 3.8, phenylalanine: 2.8, methionine: 1.9, alanine: 1.8 [56]), respectively, to select the optimum amino acid for the excipient. As shown in Figure 4, formulations with Phe added showed configuration score 2. From the result of APS measurement, formulations with Leu added showed the highest APS FPF%_≤5 μm_. The APS FPF%_≤5 μm_ values of formulations with Ile or Phe added were the second highest after Leu. Although formulations with Leu and Ile added showed high APS FPF%_≤5 μm_, these formulations showed configuration score 1 (not suitable for LDPI formulation). However, formulations with Phe added showed a high APS FPF%_≤5 μm_ and configuration score 2.

In the LDPI system, good cake appearance is necessary to ensure stable performance because LDPI formulations are aerosolized by convection flow of air in the vial. When a lyophilized cake has a poor appearance, air impact on inhalation is not equal among vials. Furthermore, the dose emitted upon inhalation can be expected to decrease due to adhesion of the cake pieces to the rubber stopper and other parts of the device. From this point, Phe, which showed both good cake appearance and high FPF (39.5 ± 3.0%), was considered as an optimum excipient for LDPI formulations of hGhrelin derivative.

#### 3.1.2. Optimization of Phe Amount in LDPI Formulation

**Figure 5:**
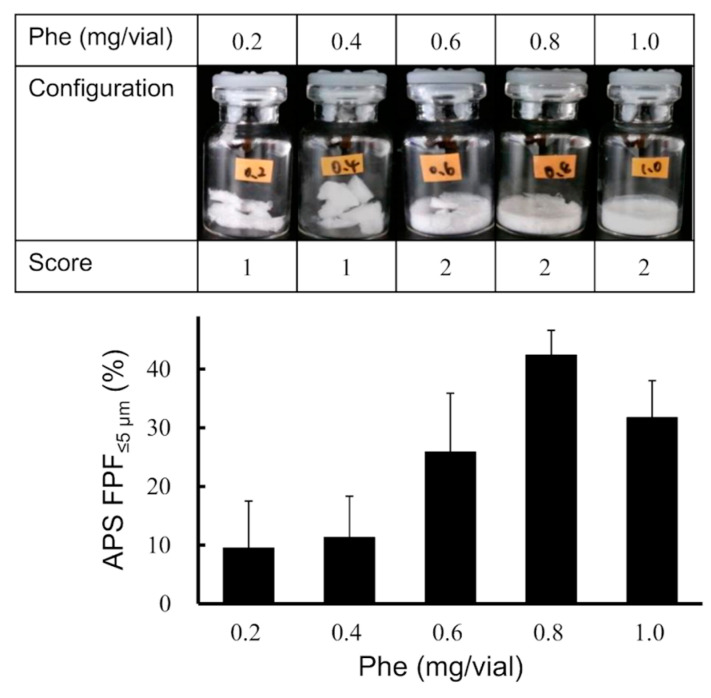
Optimization of the amount of Phe in the LDPI system. Representative photographs of formulations, configuration score, and values of APS FPF%_≤5 μm_ (mean ± SD, *n* = 3) are shown. Formulations were prepared to contain 0.5 mg/vial of hGhrelin derivative with Phe.

**Figure 6:**
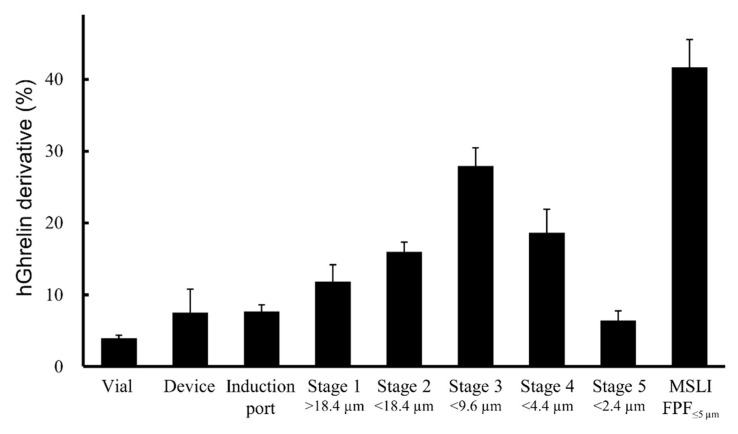
Distribution of aerodynamic particle size by MSLI in the optimal formulation of the LDPI system (mean ± SD, *n* = 3). Formulations were prepared to contain 0.5 mg/vial of hGhrelin derivative with 0.8 mg/vial of Phe.

To study the effect of the added amount of Phe on the cake appearance and aerosolization performance, formulations containing 0.5 mg/vial of hGhrelin derivative with 0.2, 0.4, 0.6, 0.8, or 1.0 mg/vial of Phe were prepared. Visual evaluation of the cake appearance and APS measurement were performed. As shown in Figure 5, formulations with 0.6, 0.8, or 1.0 mg/vial of Phe added showed configuration score 2. The APS FPF%_≤5 μm_ value of the formulation to which 0.8 mg/vial of Phe was added was the highest (42.4 ± 4.2%). Then, inhalation characteristic tests by MSLI were performed using formulations containing 0.5 mg/vial of hGhrelin derivative with 0.8 mg/vial of Phe because APS FPF%_≤5 μm_ is a surrogate benchmark for MSLI FPF%_≤5 μm_. The results of the MSLI measurements are shown in Figure 6. The MSLI FPF%_≤5 μm_ was 41.7 ± 3.8% and the MMAD was 5.6 ± 0.4 µm.

#### 3.1.3. Feasibility Evaluation of LDPI Formulation as a Preparation for PK/PD Study

**Figure 7:**
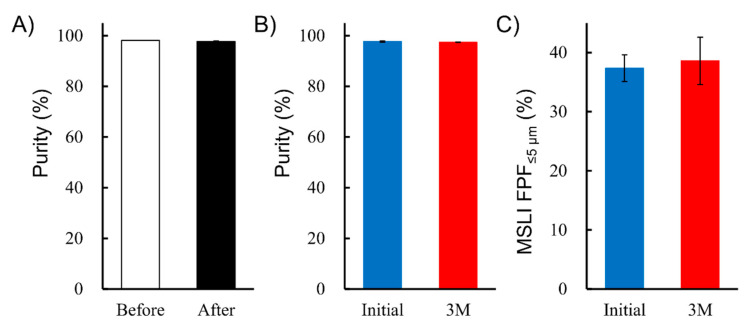
Feasibility evaluation of LDPI formulation as a preparation for PK/PD study. (**A**) Effect of lyophilization on purity of hGhrelin derivative in formulation before and after lyophilization (mean ± SD, *n* = 3). (**B**) Effect of storage (3 months at 25 °C, 60% RH) on purity of hGhrelin derivative in formulation (mean ± SD, *n* = 3). (**C**) Effect of storage (3 months at 25 °C, 60% RH) on MSLI FPF%_≤5µm_ in the LDPI system (mean ± SD, *n* = 3). Formulations were prepared to contain 0.5 mg/vial of hGhrelin derivative with 0.8 mg/vial of Phe.

To study stability in the formulation process, the purity of the hGhrelin derivative before and after lyophilization was compared. As shown in Figure 7A, there was no significant difference.

For evaluation of storage stability, formulations containing 0.5 mg/vial of hGhrelin derivative and 0.8 mg/vial of Phe were stored at 25 °C, 60% RH for 3 months. The purity of hGhrelin derivative and MSLI FPF%_≤5 μm_ was compared between before and after storage, but there were no significant differences (Figure 7B,C). This optimal formulation is thus feasible as a preparation in the PK/PD study in monkeys.

### 3.2. PK/PD Tests

To study PK/PD of the hGhrelin derivative with LDPI formulation, the optimized formulation (0.5 mg/vial of hGhrelin derivative with 0.8 mg/vial of Phe) was pulmonary administered to monkeys.

#### 3.2.1. PK Tests

**Figure 8:**
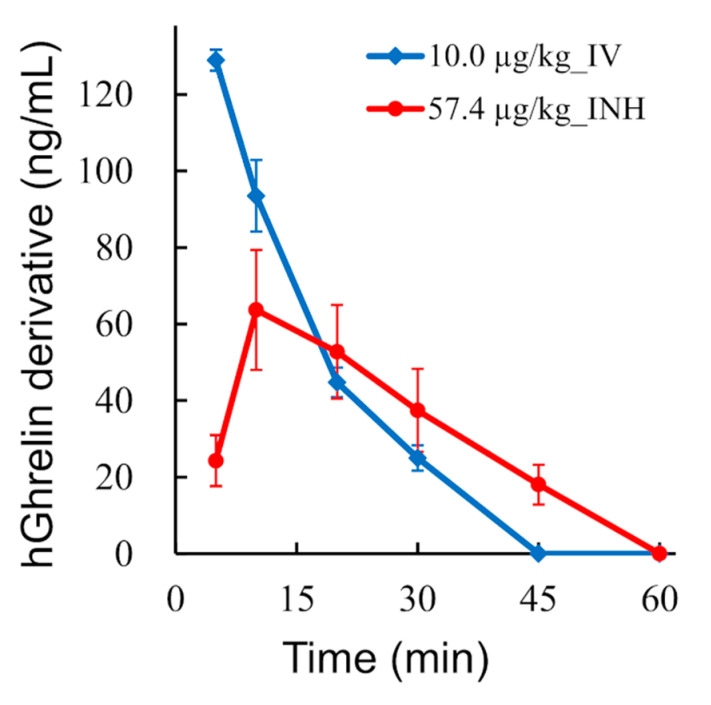
Pharmacokinetic profile of hGhrelin derivative of the LDPI system in monkeys (mean ± SE, *n* = 4). Plasma concentration of hGhrelin derivative in monkeys treated with LDPI formulation (hGhrelin derivative 57.4 µg/kg, ●) and monkeys intravenously administered hGhrelin derivative (10.0 µg/kg, ♦). IV, intravenous injection; INH, inhalation.

Formulations containing 0.5 mg/vial of hGhrelin derivative (57.4 µg/kg) were pulmonary administered, and the time profiles of the plasma concentration of hGhrelin derivative in monkeys were measured. Although the plasma concentration of hGhrelin derivative was measured from 0 to 120 min after administration, the time profiles from 0 to 60 min are shown because the concentration after 60 min was lower than the limit of quantitation. Furthermore, formulations containing 0.1 mg/vial of hGhrelin derivative (13.1 µg/kg) were pulmonary administered (*n* = 3) for comparison, but the time profiles are not shown because the plasma concentration was lower that the limit of quantitation. As shown in Figure 8, the plasma concentration of hGhrelin derivative peaked at 10 min after pulmonary administration. The area under the plasma concentration-time curve (AUC) was 1.8 ± 0.4 µg·min/mL for pulmonary administration of hGhrelin derivative (57.4 µg/kg), whereas it was 1.7 ± 0.1 µg·min/mL for intravenous administration (10.0 µg/kg). Absolute bioavailability of the inhaled hGhrelin derivative with respect to intravenous hGhrelin derivative was 16.9 ± 2.6%, assuming that the dose linearity holds for each route of administration.

#### 3.2.2. PD Tests

**Figure 9:**
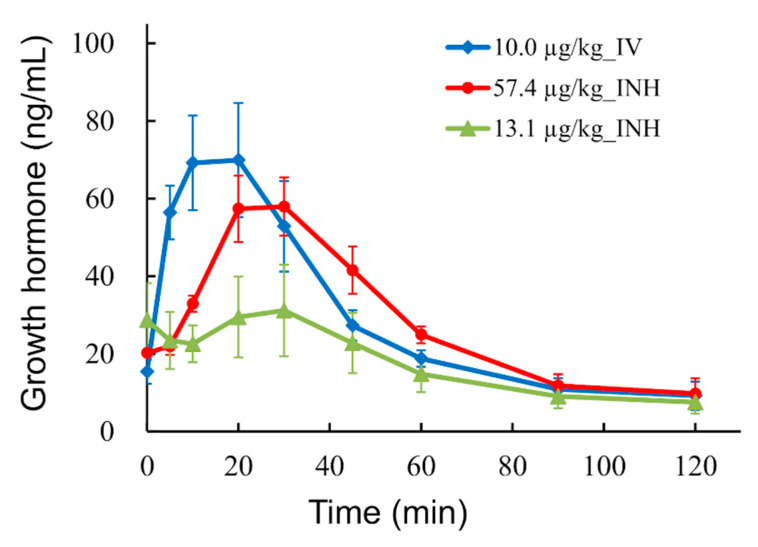
Pharmacodynamic profile of hGhrelin derivative of the LDPI system in monkeys (mean ± SE, *n* = 3 or 4). Plasma concentration of growth hormone in monkeys treated with LDPI formulation (hGhrelin derivative 57.4 µg/kg, ●; 13.1 µg/kg, ▲) and monkeys intravenously administered hGhrelin derivative (10.0 µg/kg, ♦). IV, intravenous injection; INH, inhalation.

Formulations containing 0.1 and 0.5 mg/vial of hGhrelin derivative (13.1 and 57.4 µg/kg) were pulmonary administered, and the time profiles of the plasma concentration of GH in monkeys were measured. As shown in Figure 9, the peak of the GH in plasma occurred at approximate 24 min after pulmonary administration of hGhrelin derivative (13.1 and 57.4 µg/kg). Meanwhile, an increase of GH was shown from administration (time 0) to approximately 16 min after intravenous administration of hGhrelin derivative (10.0 µg/kg). The AUCs of GH were 2.1 ± 0.6 and 3.3 ± 0.2 µg·min/mL for pulmonary administration of hGhrelin derivative (13.1 and 57.4 µg/kg), whereas it was 3.4 ± 0.5 µg·min/mL for intravenous administration.

## 4. Discussion

### 4.1. Optimization of Formulations

As a result of screening of the six amino acids, formulations with Leu added showed the highest FPF (Figure 4). Leu is known as a dispersibility enhancer excipient used in spray-drying of carrier-free DPI formulations [57,58,59]. Leu decreases the inter-particulate cohesive and adhesive forces and improves flowability and dispersibility of aerosolized particles [60,61]. The excipient showing the highest FPF value is selected as the most suitable excipient in optimization of the formulation for conventional DPIs because aerosolization performance is the key characteristic. However, the LDPI formulation is prepared as not particles but a lyophilized cake as shown in Figure 1. In the LDPI system, a lyophilized cake is aerosolized just upon inhalation by air impact. When the cake appearance is poor, aerosolization performance on inhalation may fluctuate due to static generation among fragmented pieces of the cake [62]. In other words, good cake appearance is necessary to guarantee stable aerosolization performance of LDPI formulations. Thus, excipients that show both high FPF and good cake appearance should be selected as the suitable excipient in optimization of LDPI formulations. Lyophilizate of an LDPI formulation is broken into pieces by air impact and aerosolized by the convection flow of air in the vial. Then, porous particles reconstructed from pieces of lyophilizate are emitted through the device. When a lyophilized cake has a poor appearance, air impact is diffused, which may cause insufficient disintegration of the lyophilized cake, and air impact on inhalation is not equal among vials. Furthermore, aerosolization performance may be non-constant because a static electrical charge is generated among fragmented pieces of lyophilizate. In addition, the dose emitted upon inhalation can be expected to decrease due to adhesion of the cake pieces to the rubber stopper and other parts of the device. Thus, excipients for LDPI formulations require both good cake appearance and high FPF. In general, good cake appearance and high FPF are not compatible. In practice, formulations with Leu added showed poor cake appearance with high FPF (Figure 4). Therefore, leucine was considered to be undesirable as an excipient for LDPI formulations. From the above, we selected phenylalanine as the excipient to optimize LDPI formulations of the hGhrelin derivative.

Phe showed both good cake appearance and high FPF. In the screening study, the APS FPF%_≤5 μm_ value of the formulation with Phe added was approximately 40% (Figure 4). In addition, formulations with Phe added showed higher FPF when the cake appearance was good than when the cake appearance was poor (Figure 5). In the LDPI system, it is considered that hydrophobic amino acids show high aerosolization performance due to their surface activity. Unlike saccharides, hydrophobic amino acids with surface activity tend to coordinate at the ice/water and air/water interfaces during freezing, thus forming a hydrophobic coating film that covers the porous matrix structure of the lyophilized cake. This film may cause the high aerosolization performance. Phe has higher surface activity than other hydrophobic amino acids [63] and is an aromatic amino acid. Presence of a side chain with aromatic ring is considered one of the reasons for good cake appearance. An aromatic side chain was reported to show higher glass transition temperature due to the stacking interaction [64]. High glass transition temperature is one of the important factors that contributes to good cake appearance [53]. Therefore, it is considered that Phe may show both good cake appearance and high FPF due to its aromatic side chain and surface activity.

As a result of the study of the effect of the added amount of Phe, the optimized formulation of hGhrelin derivative (0.5 mg/vial) was prepared with 0.8 mg/vial of Phe (Figure 5). MSLI FPF%_≤5 μm_, an index of the efficiency at which a formulation can reach the lungs, of the optimized formulations was approximately 40% (Figure 6). The difference between MSLI FPF%_≤5 μm_ and APS FPF%_≤5 μm_ was within 1.0%. Our simple method using APS (Figure 2) was shown to be useful as a surrogate method for MSLI to optimize a formulation in a short time.

The stability tests showed no significant difference in the purity of the hGhrelin derivative assessed before and after lyophilization (Figure 7A). This means that LDPI can solve the problem of degradation and denaturation due to shear stress or high temperature during the manufacturing process of conventional DPIs, as expected.

### 4.2. Prospect for Clinical Application

The optimized formulation for PK/PD (0.5 mg/vial of hGhrelin derivative with 0.8 mg/vial of Phe) was pulmonary administered to monkeys and pulmonary absorption was evaluated. In vivo models of large animals are desirable to study pulmonary PK with formulation and/or device efficiency for prediction of the human PK profile [65,66]. As pulmonary administration methods for animals, intratracheal instillation or aerosol exposure is commonly used [66,67]. In the aerosol exposure method, the dose determination and absorption from extra-lung (nose and gastrointestinal tract) should be a concern in the PK analysis [68]. In contrast, intratracheal instillation can deliver a controlled quantity of drug directly into the lungs [66]. The use of sprayers such as the Pen Century dry powder insufflator are known as a method to administer powder aerosols of DPI intratracheally [69,70]. In the method using a sprayer, powders are loaded into the chamber of an insufflator device and delivered with additional air. However, the LDPI formulation consists not of particles but of a freeze-dried cake. Hence, we made a device to administer LDPI formulations for human clinical use intratracheally to monkeys so that pulmonary absorption with the efficiency of LDPI formulations could be evaluated.

As a result of the PK tests, the absolute bioavailability of inhaled hGhrelin derivative with respect to intravenously injected hGhrelin derivative was calculated to be about 17% assuming that the dose linearity holds for each route of administration. The absolute bioavailability of the Afrezza^®^ (insulin inhaler) was 14.7% in human [71]. Pulmonary absorption of hGhrelin derivative with the LDPI system was expected to be comparable with a commercial DPI for systemic delivery.

In the PD tests, the time profiles of the plasma concentration of GH in monkeys were measured. Plasma GH increase is one of the markers used to detect the effect of ghrelin through GHSR [5,72]. The AUC of GH for inhalation (hGhrelin derivative 57.4 µg/kg) roughly agreed with that for intravenous injection. However, the onset time of the GH increase was approximately 10 min later when hGhrelin derivative was pulmonary administered rather than intravenously injected (Figure 9). Inhaled hGhrelin derivative was absorbed through the lungs and then penetrated the systemic circulation leading to GH release. The plasma concentration of hGhrelin derivative peaked at 10 min after pulmonary administration (Figure 8), and the peak time was equal to the delay time of the GH increase. Thus, the delay of GH increase was considered to be due to absorption and distribution of the inhaled hGhrelin derivative. Comparing the AUCs of GH for inhalation, the AUC was about 1.6 times higher for 57.4 µg/kg of hGhrelin derivative administered than for 13.1 µg/kg. Although GH release increased depending on the administered dose of hGhrelin derivative, the difference in the GH AUC by administered dose was smaller than expected. There are two possible reasons for this result. One is the ceiling effect. In previous studies [19,73,74], the AUC of GH in healthy humans was about 1.3–1.6 times higher when 5 µg/kg of ghrelin was intravenously injected versus 1 µg/kg because of the ceiling effect. The other is physical stress. GH is known to be secreted in response to physical stress [75]. It is undeniable that physical stress was involved in the GH increase after pulmonary administration of hGhrelin derivative, but it was considered that the GH increase was mainly caused by inhaled hGhrelin derivative based on the onset time delay and AUC ratio.

From the results of the PK/PD tests, LDPI formulations of hGhrelin derivative may be expected to be used for systemic application by the pulmonary route.

## 5. Conclusions

In this study, we conducted PK/PD tests in monkeys using a LDPI formulation to evaluate its feasibility for systemic delivery of hGhrelin derivative. LDPI formulations for PK/PD tests were optimized by the addition of Phe (0.5 mg/vial of hGhrelin derivative with 0.8 mg/vial of Phe). The MSLI FPF%_≤5 μm_ value of the formulation used in the PK/PD tests was 41.7 ± 3.8%. In the PK tests, the AUC was 1.8 ± 0.4 µg·min/mL for pulmonary administration of hGhrelin derivative (57.4 µg/kg), whereas it was 1.7 ± 0.1 µg·min/mL for intravenous administration (10.0 µg/kg). In the PD tests, the AUCs of GH were 2.1 ± 0.6 and 3.3 ± 0.2 µg·min/mL for pulmonary administration of hGhrelin derivative (13.1 and 57.4 µg/kg), whereas it was 3.4 ± 0.5 µg·min/mL for intravenous administration. GH release increased depending on the dose of hGhrelin derivative, although the difference in AUCs was smaller than expected. Furthermore, the onset time of the GH increase was approximately 10 min later when hGhrelin derivative was pulmonary administered rather than intravenously injected, and the delay time of the GH increase was equal to the peak time of hGhrelin derivative by pulmonary administration. It was considered that inhaled hGhrelin derivative was absorbed through the lungs and then penetrated the systemic circulation leading to GH release. From the above, LDPI formulations of hGhrelin derivative may be expected to be used for systemic application by the pulmonary route to treat diseases such as eating disorders and cachexia.

## Data Availability

The data presented in this study are available on request from the corresponding author.

## Supplementary Materials

The following are available online at https://www.mdpi.com/1999-4923/13/2/233/s1, Figure S1: Chemical structure of hGhrelin derivative.
