# Supplementary material for: Systemic Delivery of hGhrelin Derivative by Lyophilizate for Dry Powder Inhalation System in Monkeys
Source: Pharmaceutics. 2021 Feb 7;13(2):233. doi: 10.3390/pharmaceutics13020233 (PMC7914841; doi:10.3390/pharmaceutics13020233)
Supplement: Supplementary file 1 [file pharmaceutics-13-00233-s001.pdf]

pharmaceutics MDPI

Supplementary Materials: Systemic Delivery of hGhrelin Derivative by Lyophilizate for Dry Powder Inhalation System in Monkeys

Kahori Miyamoto, Yuko Ishibashi, Tomomi Akita and Chikamasa Yamashita

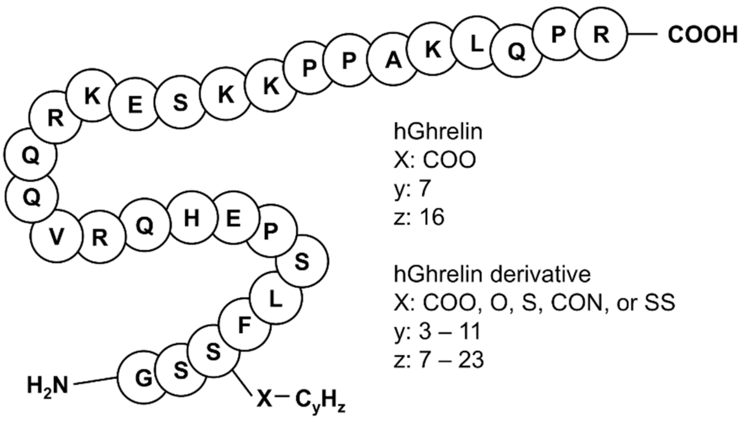

Figure S1: Chemical structure of hGhrelin derivative.

Activity of human ghrelin (hGhrelin) is expressed by *O*-*n*-octanoylation at the 3rd serine residue, but the ester bond is easily cleaved in blood plasma. The ester bond or the lipidic regions of hGhrelin derivative are modified so that it is less likely to be des-acylated. A, Alanine; E, Glutamic acid; G, Glycine; F, Phenylalanine; H, Histidine; K, Lysine; L, Leucine; P, Proline; Q, Glutamine; R, Arginine; S, Serine; V, Valine.
